# Massive Device-Related Thrombus After Left Atrial Appendage Closure in Kidney Transplant Recipient With Hypertrophic Cardiomyopathy

**DOI:** 10.1016/j.jaccas.2026.107254

**Published:** 2026-03-11

**Authors:** Tianyang Lu, Guanhua Wang, Sha Zhang, Yongwen Qin, Zhifu Guo, Yuan Bai

**Affiliations:** Department of Cardiology, Changhai Hospital, Naval Medical University, Shanghai, P.R. China

**Keywords:** anticoagulation, device-related thrombus, hypertrophic cardiomyopathy, left atrial appendage closure

## Abstract

**Background:**

Although left atrial appendage closure (LAAC) provides a crucial stroke prevention alternative for patients with atrial fibrillation (AF) who cannot undergo long-term anticoagulation, concurrent hypertrophic cardiomyopathy (HCM) elevates the risk of device-related thrombosis (DRT) after the procedure.

**Case Summary:**

This case report describes a 59-year-old woman with HCM, permanent AF, and a history of renal transplantation. Stroke prophylaxis was performed via LAAC with a 33-mm Watchman device. Premature discontinuation of dual-antiplatelet therapy 1 month postprocedure led to a large (5.2 cm × 2.5 cm) DRT identified at 6-month follow-up. The thrombus resolved completely after a prolonged, staged, and dose-adjusted course of rivaroxaban.

**Discussion:**

The efficacy and safety of LAAC for stroke prevention in patients with HCM and AF who cannot tolerate anticoagulation remain debated

**Take-Home Message:**

Although LAAC is not a standard stroke prevention therapy for HCM patients with AF, it represents a viable alternative in high-bleeding-risk scenarios and requires meticulous planning, vigilant DRT surveillance, and individualized antithrombotic management.

## Visual Summary

**Figure:**
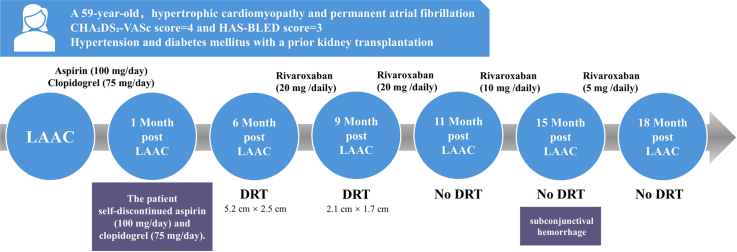

## History of Presentation

A 59-year-old woman presented with recurrent palpitations for over 5 years and was found to have permanent atrial fibrillation.

**Take-Home Message**

• Although left atrial appendage closure is not a standard stroke prevention therapy for hypertrophic cardiomyopathy patients with atrial fibrillation, it represents a viable alternative in high-bleeding-risk scenarios and requires meticulous planning, vigilant device-related thrombosis surveillance, and individualized antithrombotic management.

## Past Medical History

She had a history of hypertension and diabetes mellitus and had previously undergone kidney transplantation for end-stage kidney disease.

## Differential Diagnosis

In this patient with an elevated bleeding risk (HAS-BLED score 3) and on long-term immunosuppressants after renal transplantation, the addition of anticoagulation carries a significant risk of hemorrhage secondary to pharmacokinetic interaction. Consequently, left atrial appendage closure (LAAC) was proposed as a nonpharmacological alternative to warfarin for stroke prophylaxis.

## Investigations

Before LAAC, transesophageal echocardiography (TEE) confirmed the absence of contraindications to the procedure, including active intracardiac thrombus and any condition requiring lifelong anticoagulation therapy, such as the presence of a mechanical heart valve or history of pulmonary embolism. Cardiac imaging demonstrated segmental left ventricular hypertrophy with apical predominance, characterized by significant septal thickening (16 mm) and mild posterior wall thickening (13 mm). The typical “ace of spades” morphology was observed at the apex. No left ventricular outflow tract obstruction was present under resting or provocation.

## Management

**Figure 1:**
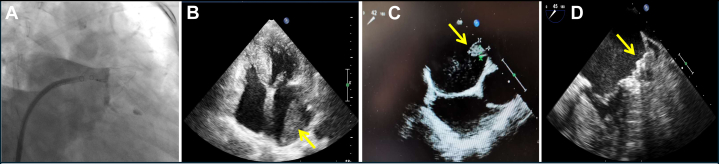
Device-Related Thrombosis Visualization and Removal (A) Successful implantation of a 33-mm Watchman occluder with no detectable peridevice leaks. (B) A left atrial thrombus (5.2 cm × 2.5 cm) was revealed on transthoracic echocardiography (TTE) at the 6-month follow-up. (C) The thrombus (2.1 cm × 1.7 cm) was shown on transesophageal echocardiography (TEE) at the 9-month follow-up (3 months after initiating anticoagulation). (D) Sustained complete thrombus resolution was demonstrated on TEE at the 18-month follow-up (12 months after initiating anticoagulation).

LAAC was performed under general anesthesia via right femoral vein access. Left atrial appendage angiography revealed a cauliflower left atrial appendage with multiple lobes and showed that the diameter of the landing area was 29 mm. A 33-mm Watchman occluder (Boston Scientific) was implanted with adequate compression of 24% and no detectable peridevice leaks. (Figure 1A).

## Outcomes and Follow-Up

Dual-antiplatelet therapy with aspirin (100 mg/d) and clopidogrel (75 mg/d) was then prescribed. After LAAC, the patient received the medication for 1 month; however, its use was not continued thereafter. At 6-month postoperative follow-up, transthoracic echocardiography (TTE) revealed a large left atrial thrombus measuring 5.2 cm × 2.5 cm (Figure 1B, Video 1). Anticoagulation therapy with oral rivaroxaban (20 mg/d) was initiated immediately thereafter. Follow-up TEE at 9 months postprocedure (3 months after anticoagulation) showed reduction of the thrombus to 2.1 cm × 1.7 cm (Figure 1C). By 11-month postoperative follow-up (5 months of anticoagulation), complete thrombus resolution was confirmed on TTE, and the rivaroxaban dose was adjusted to 10 mg/d. At 15 months postprocedure, no thrombus recurrence was observed on TTE; however, because of the occurrence of subconjunctival hemorrhage, the dose was further reduced to 5 mg/d. Ultimately, complete thrombus resolution was sustained and confirmed at 18-month postoperative follow-up with TEE (Figure 1D, Video 2).

## Discussion

**Figure 2:**
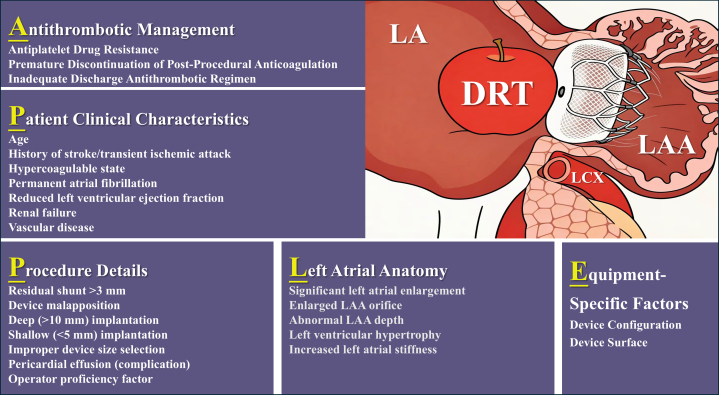
“APPLE” Framework: Risk Factors for Device-Related Thrombosis The “APPLE” framework provides a structured way to remember that device-related thrombosis (DRT) risk stems from a combination of medication management, patient comorbidities, procedural technique, anatomical features, and device characteristics. A = *a*ntithrombotic management: examples include antiplatelet drug resistance (eg, clopidogrel), premature discontinuation of postprocedural anticoagulation, and inadequate discharge antithrombotic regimens; P = *p*atient clinical characteristics: examples include advanced age, history of stroke/transient ischemic attack, hypercoagulable state, permanent atrial fibrillation, reduced left ventricular ejection fraction, renal failure, and vascular disease; P = *p*rocedure details: examples include residual shunt >3 mm, device malapposition (incomplete seal), deep implantation, improper device size selection, pericardial effusion, and operator proficiency; L = *l*eft atrial (LA) anatomy: examples include significant LA enlargement, abnormal left atrial appendage (LAA) morphology (eg, chicken wing and wind sock), left ventricular hypertrophy, and increased LA stiffness; E = *e*quipment-specific factors: examples include device configuration and device surface. LCX = left circumflex artery.

The evolving landscape of stroke prevention in atrial fibrillation has witnessed a growing prominence of percutaneous LAAC, particularly as a viable alternative for patients with contraindications to long-term oral anticoagulation.^1^ The incidence of device-related thrombosis (DRT) after LAAC is approximately 3.8%, presenting a major concern during follow-up. Patients with DRT have a significantly elevated risk of ischemic events, approximately 4 to 5 times higher than that in those without DRT (ischemic event rates: 13.2% in DRT patients vs 3.8% in non-DRT patients).^2^ DRT risk factors can be summarized by the “APPLE” framework, encompassing 5 dimensions: *antithrombotic management* (eg, antiplatelet drug resistance, premature discontinuation of postprocedural anticoagulation, and inadequate discharge antithrombotic regimens), *patient clinical characteristics* (eg, advanced age, history of stroke/transient ischemic attack, hypercoagulable state, permanent atrial fibrillation, reduced left ventricular ejection fraction, renal failure, and vascular disease), and *procedure details* (eg, residual shunt >3 mm, device malapposition, deep implantation, improper device size selection, pericardial effusion, and operator proficiency). Additional contributors include *left atrial anatomy* (eg, significant enlargement, abnormal left atrial appendage morphology, left ventricular hypertrophy, and increased left atrial stiffness) and *equipment-specific factors* related to device configuration and surface properties^2^, ^3^, ^4^, ^5^ (Figure 2).

Notably, in the subset of patients with hypertrophic cardiomyopathy (HCM), LAAC exhibits a distinct risk-benefit paradox: it confers a relative reduction in bleeding risk without adversely affecting mortality, yet it is linked to an elevated propensity for DRT, ischemic stroke, and systemic thromboembolism.^6^ This necessitates a carefully individualized risk assessment in this population. This is an extremely rare case of massive left atrial thrombus after LAAC in an HCM patient. In addition to renal impairment and the cessation of dual-antiplatelet therapy, this patient with HCM exhibited a cluster of typical high-risk features for DRT, such as hypercoagulability, postoperative pericardial effusion, nonparoxysmal atrial fibrillation, pronounced left ventricular hypertrophy, and an enlarged left atrial appendage ostium. According to current guidelines, LAAC is not indicated as a first-line stroke prevention strategy for patients with HCM and concomitant atrial fibrillation.^7^ However, with ongoing investigative efforts, the incidence of DRT has shown a steady decline. Beyond the steady decline in DRT incidence achieved through ongoing device refinement, recent efforts have incorporated computational fluid dynamics–based virtual implantation to predict patient-specific hemodynamic stagnation zones across different deployment scenarios, thereby guiding optimal implantation planning and addressing DRT risk at its hemodynamic source.^4^

Available data indicate that extended administration of reduced-dose direct oral anticoagulants post-LAAC is associated with a significant decrease in the incidence of DRT, thromboembolic events, and major hemorrhage.^8^ A meta-analysis found that in patients undergoing LAAC, compared with standard dual-antiplatelet therapy, the use of low-dose direct oral anticoagulants postprocedure more effectively reduced the risk of thromboembolic events, including DRT, while also resulting in fewer bleeding events and demonstrating better safety. This suggests that low-dose direct oral anticoagulants are a promising and potentially superior option for anticoagulation management after LAAC, particularly in patients with high thrombotic and bleeding risks.^9^ These observations suggest that sustained low-intensity anticoagulation could serve as a viable management option after LAAC for high-risk patients with concurrent bleeding and thromboembolic susceptibility, including patients with HCM who have a high risk of bleeding. Although current evidence does not support the use of LAAC as a complete alternative to oral anticoagulation for patients with HCM and atrial fibrillation, future advancements in device technology and procedural techniques may position it as a viable option in selected cases.

**Visual Summary:**
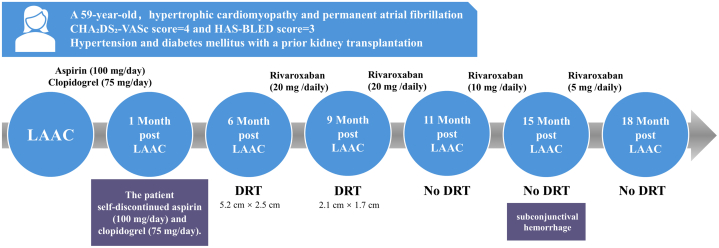
Chronology of Clinical Follow-Up A 59-year-old woman with hypertrophic cardiomyopathy, permanent atrial fibrillation, and a history of renal transplantation underwent left atrial appendage closure (LAAC) with a 33-mm Watchman device. During scheduled follow-ups at 6, 9, 11, 15, and 18 months postprocedure, a large left atrial thrombus (5.2 cm × 2.5 cm) was detected at the 6-month evaluation after the patient's self-discontinuation of dual-antiplatelet therapy 1 month after LAAC. The thrombus was successfully managed with a prolonged (>12 months), dose-titrated course of rivaroxaban, leading to complete resolution. DRT = device-related thrombosis.

## Conclusions

This case highlights the current need for particularly cautious evaluation of LAAC in atrial fibrillation patients who have comorbid HCM and documented long-term anticoagulation intolerance.

## Funding Support and Author Disclosures

This work is a subproject of the National Key Research and Development Program of China, administered by the China National Center for Biotechnology Development (2023YFC2412700). The authors have reported that they have no relationships relevant to the contents of this paper to disclose.

## Appendix

Video 1 Large Left Atrial Thrombus Detected 6 Months Post-LAAC

Video 2 Sustained Thrombus Resolution at 18-Month TEE Follow-Up
